# Risk factors for measles mortality and the importance of decentralized case management during an unusually large measles epidemic in eastern Democratic Republic of Congo in 2013

**DOI:** 10.1371/journal.pone.0194276

**Published:** 2018-03-14

**Authors:** Etienne Gignoux, Jonathan Polonsky, Iza Ciglenecki, Mathieu Bichet, Matthew Coldiron, Enoch Thuambe Lwiyo, Innocent Akonda, Micaela Serafini, Klaudia Porten

**Affiliations:** 1 Epicentre, Paris, France; 2 Médecins Sans Frontières, Geneva, Switzerland; 3 Médecins Sans Frontières, Paris, France; 4 Ministère de la Santé Publique, Kinshassa, République Démocratique du Congo; Universidade Nova de Lisboa Instituto de Higiene e Medicina Tropical, PORTUGAL

## Abstract

In 2013, a large measles epidemic occurred in the Aketi Health Zone of the Democratic Republic of Congo. We conducted a two-stage, retrospective cluster survey to estimate the attack rate, the case fatality rate, and the measles-specific mortality rate during the epidemic. 1424 households containing 7880 individuals were included. The estimated attack rate was 14.0%, (35.0% among children aged <5 years). The estimated case fatality rate was 4.2% (6.1% among children aged <5 years). Spatial analysis and linear regression showed that younger children, those who did not receive care, and those living farther away from Aketi Hospital early in the epidemic had a higher risk of measles related death. Vaccination coverage prior to the outbreak was low (76%), and a delayed reactive vaccination campaign contributed to the high attack rate. We provide evidences suggesting that a comprehensive case management approach reduced measles fatality during this epidemic in rural, inaccessible resource-poor setting.

## Introduction

Since 1990, measles incidence has significantly decreased in every region of the world[1]. However, this progress has been mitigated since 2007, most of the cases have been reported in sub-Saharan Africa [2][3]. The Democratic Republic of Congo (DRC) has followed this trend: in 2009, only 3364 cases were reported, a minimum for the country, but since 2010, a prolonged epidemic has affected the country, and the number of cases has substantially increased. Nationwide, 134 041 cases were reported in 2011, 73 794 in 2012 and 87 365 in 2013. [4] [5].

Following WHO recommendations for measles mortality reduction settings[6], DRC’s national strategy for measles control relies on the Expanded Program on Immunization (EPI), including routine immunization, regular supplementary immunization activities (SIAs), and Outbreak Response Immunization (ORI) once an epidemic has been confirmed[7].

The Province Orientale is a vast region in northeastern DRC, where access to health care provision is often limited due to poor infrastructure, long distances, and insecurity. A measles epidemic in the province was declared during week 18 of 2012, with many local epidemics declared during the following year. The international medical humanitarian organization Médecins Sans Frontières (MSF) supported the Ministry of Health (MoH) in its response to the outbreak.

The Aketi Health Zone (HZ), with an estimated population of 128 892, is situated in the Bas-Uele District of the Province Orientale. Between January 2006 and June 2012, a total of 8 suspected measles cases were declared in the HZ, never more than one per week. A SIA targeting all children aged 6 months to 15 years was conducted in September 2011.

**Fig 1:**
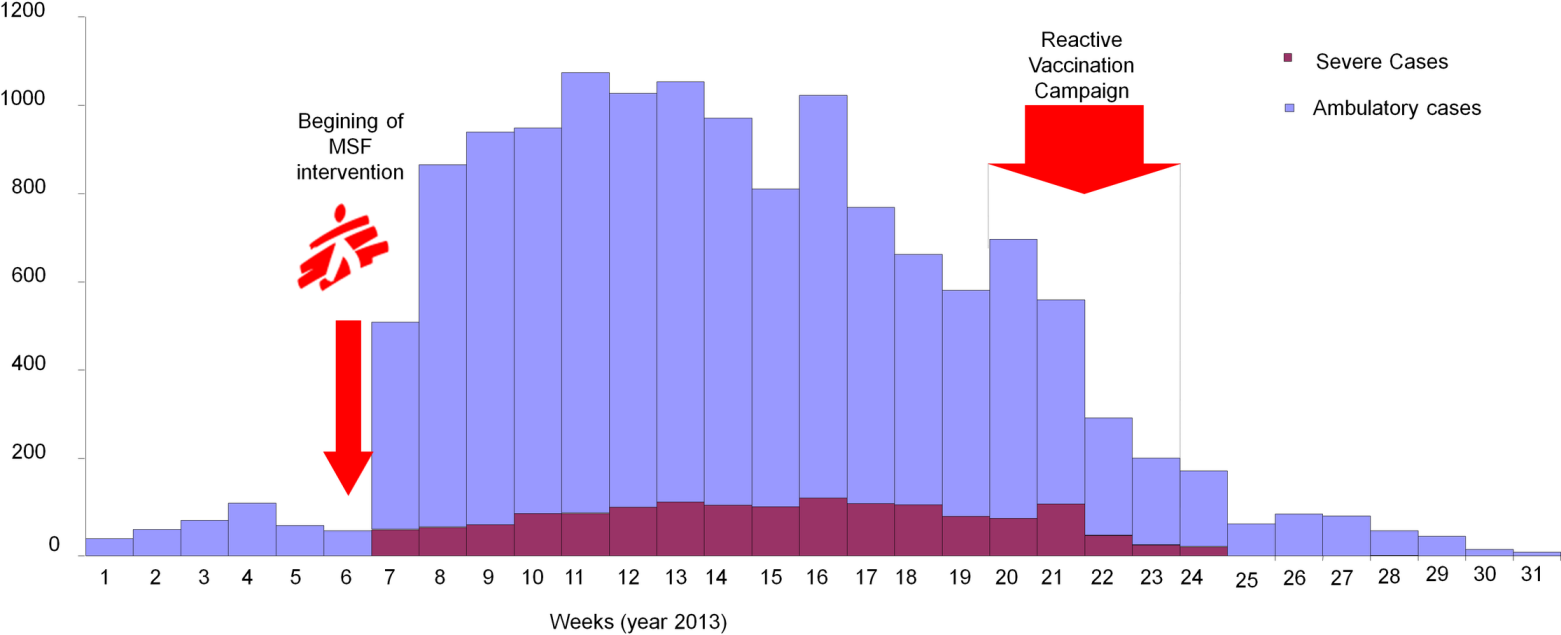
Suspected measles cases by week, Aketi Health Zone, Province Orientale, DRC, 2013 (source: MSF and MoH).

Between July and December 2012, 48 suspected measles cases were reported in Aketi HZ. Then, between January and July 2013, 13 413 suspected cases were reported (attack rate 10.4%); 1 633 cases were hospitalized, 23 measles-related deaths were also reported, corresponding to a reported Case Fatality Ratio (CFR) of 0.2%. (Fig 1). In response to the increase in cases, MSF started providing direct support to the MoH in Aketi in February 2013. A measles epidemic was officially declared in the HZ on April 17, with an ORI starting May 13^th^ and ending June 6^th^ , after the epidemic had already reached its peak.

The MSF intervention supporting the MoH included the following:

Access to free health care for measles patients at all levels of health care (from health post to hospital); incentives were given to MOH staff involved in treatment and a monthly fee was paid to health facilities to compensate for loss of income;
Direct support to the intensive care unit of Aketi Hospital;
Continuous training, regular supply of drugs (treatments including vitamin A were made available and free for every suspected measles cases), collection of data, and regular supervision in peripheral health centers and health posts;
A free motorbike referral system from peripheral health structures to Aketi Hospital;
Active case finding by Community Health Workers.

Due to the unusually high reported attack rate with relatively low CFR, we conducted a population-based survey.

In the result section, the sub-section “description of the epidemic” present the estimate of measles attack rates, measles-specific mortality during the epidemic and the vaccination coverage; in the sub-section “risk factors for measles mortality, we explore the potential risk factors associated with the measles fatality through logistic regression and geospatial analysis. In the discussion section, we remind the burden caused by a measles epidemic, we discuss the effectiveness of a comprehensive case management system where the combination of decentralized health care and referral seems to have reduced significantly the case fatality; finally, we report the weakness of the implementation of the prevention and control measures.

## Material and methods

### Design and sample size

We used two-stage cluster sampling which is an efficient design to cover a large population in a short time frame with limited number of surveyors[8]. Clusters were randomly selected from a list of villages and neighborhoods with probability proportional to population size (based upon estimates provided by the health authorities). Households were selected following the method proposed in the World Health Organization ‘s manual : Immunization coverage cluster survey–Reference manual [8]. The first household of each cluster was selected randomly. In the absence of a household list, a random direction was selected from the approximate centre of the chosen village. All households were then counted from the centre to the edge of village. A number was randomly selected (between one and the number of counted households). This number represented the first selected household. Subsequent households were selected by proximity (nearest household to the left). Heads of household (or their representatives) were interviewed using a questionnaire modeled on a questionnaire developed by WHO to investigate measles CFR [9]. The following information was collected for each person who met the criteria for having been a household member during the recall period: age and sex; death during the recall period (including reported date, cause and location of death); measles vaccination history (according to a document or parental recall); and whether the person had demonstrated measles-like symptoms during the recall period. For each reported suspected measles case, the following additional information was collected: symptoms, date of illness, and the type of treatment received.

Additionally, GPS coordinates were taken at the center of each cluster included, and the distances by footpath from the cluster to the nearest health post, health center and hospital were collected.

The sample size was calculated to estimate measles-specific mortality rate. The assumptions used for the calculation were an expected measles-specific mortality of 1 per 10 000 person-days, with a precision of ±0.5, a recall period of 288 days, a non-response rate of 3%, a design effect of 1.5 and an average household size of 5. The sample size necessary was calculated to be 7105 individuals using ENA software [10]. We therefore selected 40 clusters of 36 households each.

### Case definition, recall period and study population

The case definition for measles followed WHO recommendations [11]: i.e. any person with a generalized maculo-papular rash and a history of fever of 38°C or more (or “hot to touch” if not measured) and at least one of the following: cough, coryza, or conjunctivitis. A measles death was any death during the 28 days following the onset of measles. [9]

The recall period ran from 25 December 2012 (Christmas day being a well-known date in the study area) until the date of interview (between 9 and 13 October 2013). All persons living in the Aketi HZ at any point during the recall period were included in the target population.

Because of difficult access (more than one full day of transport by motorbike, bicycle or walking), several villages were excluded from the original sampling frame. The population of these villages was estimated at 5.1% of the total population of Aketi HZ.

### Statistical analysis

Attack rates, case fatality rates and mortality rates were estimated, under the assumption that the sample was self-weighting. We calculated 95% confidence intervals and design effects for each estimate using a Taylor linearized variance estimation. We estimated the total number of death caused by measles during the outbreak in the entire health zone by applying the measles specific mortality rates found in the survey to the total population (128 892 inhabitants). To estimate the measles related death in children under 5 years old, we applied the age specific measles mortality rate of this age group to the total population multiplied by the proportion of children of this age group found in the survey.

To describe the risk factors associated with measles mortality, we fitted a log Poisson regression model. We used Poisson regression which is appropriate to model count data (number of death in our case). We used a multilevel model including random effects at cluster and household level to distinguish factors that are measured at cluster, household and individual level [12]. All variables collected during the interview were included in the model, and the distance to the nearest health center or health post (in kilometers). We considered that 30 km distance is the maximum distance a caretaker could walk during one day; therefore, we included the distance to the hospital as a categorical variable (< = 30 km vs. >30km). As access to, and quality of, health care may have changed because of the MSF support described above, a dichotomous variable was additionally created to distinguish those cases reported before and after 1 March, when the intervention became fully operational.

In univariate analysis, all variables listed above were tested for association with measles mortality. Variables with p<0.2 in univariate analysis were included in the multivariate analysis. Because a majority of measles deaths were reported at the periphery of the health zone and before March 1, we also explored the effect of an interaction term between the period (before or after March 1^st^) and the distance to the hospital (more or less than 30 km), we applied linear combinations to present the main effects and the effect caused by the interaction. The regression model used stepwise forward selection; we added one by one every variables included in the univariate analysis starting by the variable with the lowest p value, variables were retained when p<0.05 using the Wald test.

We also conducted geospatial analyses of the measles CFR for the periods prior to and during the MSF intervention. The R package ‘Spatstat’ was used to create point pattern datasets, applying cluster-specific estimates of CFR to coordinates of each cluster. We then used a Gaussian kernel function to smooth this data, and reported the results in heat maps [13][14]. Survey data were entered in Excel (Microsoft, Redmond, WA, USA) and analyzed in Stata v11 (college Station, Texas, USA), spatial analysis were performed with R statistical software.

### Ethical considerations

The procedures conducted were in accordance with the ethical standards of the Helsinki Declaration. The survey was conducted after obtaining authorization from provincial health authorities of Lubumbashi in Democratic Republic of Congo. The study is considered as program monitoring by Médecins Sans Frontières. An informed consent statement was read aloud in the local dialect and interviews were only conducted if verbal consent was given by the household head; it was clearly stated that participants were free to withdraw from the study at any time. The consent or refusals was recorded. Verbal consent was chosen over written consent to avoid potential issues with literacy, due to the low-risk nature of the study and because personal information such as names or other identifiers was not recorded. Interviews were conducted by 12 surveyors under the direct supervision of the principle investigator and two deputies. Surveyors were trained before the survey on the methods and ethics rules to conduct interview for epidemiological research.

## Results

From 9 to 13 October 2013, among the 1,440 households targeted, 1,424 households were interviewed (non-response rate: 1.1%) representing 8054 people, among whom 1,482 were aged <5 years. The mean household size was 5.7 members. 192 persons joined, and 175 persons left, the households during the recall period. 122 deaths and 244 births were reported as occurring during the recall period (birth rate: 40.0 birth/1000 person-years). The overall male to female ratio (M/F) was 0.95.

### Description of the epidemic

#### Attack rate

A total of 1131 persons met the measles case definition during the recall period, corresponding to a crude AR of 14.0% (95% CI 12.2–15.8; Table 1). Every cluster reported measles cases (median number of cases per cluster: 27, range 3–63). 1131 cases were reported from 603 households with an average household size of 6.6 individuals, among which on average 1.9 measles cases were reported.

Among children aged <5 years, the measles-specific AR was 35.0% (95% CI 31.3–38.8).

**Table 1:** Measles attack rate by age, December 25, 2012 to October 9, 2013, Aketi Health Zone, Province Orientale, Democratic Republic of Congo.

Age at time of the survey	Suspected Measles cases	Attack rate(%)	95% CI	Design effect	ICC*
0–11 months	53	14.9%	8.2–21.6	3.3	0.292
12–23 months	104	45.6%	36.1–55.0	2.1	0.234
24–59 months	362	40.2%	35.6–44.9	2.1	0.051
5–15 years	419	18.3%	14.9–21.7	4.7	0.066
> 15 years	189	4.4%	3.1–5.6	4	0.028
Unknown	4	-	-	-	
**Total**	**1131**	**14.0%**	**[12.2–15.8]**	**5.8**	**0.024**

* ICC is calculated as follows: (design effect– 1)/(average cluster size– 1)

### Specific mortality rate and case fatality rate

During the recall period, 48 deaths were attributed to measles (including 37 among children <5), corresponding to an estimated measles-specific mortality rate of 0.25 deaths per 10000 person-days (95% CI 0.16–0.34). Among children aged <5 years the estimated measles-specific mortality was 0.98 per 10000 person-days (95% CI 0.63–1.32).

Interpolating this measles-specific mortality rate over the entire population of the Aketi HZ leads to an estimate of 798 deaths attributable to measles (95% CI: 520–1080) during the recall period, including 669 among children <5 (95% CI: 430–905).

The overall estimated CFR for measles was 4.2% (CI 95%: 2.8–5.7) and among children <5 at time of the disease, it was 6.1% (CI 95%: 4.0–8.2).

### Reported measles vaccination coverage

When considering both the routine immunization and the SIA of 2011, overall measles vaccination coverage (at least one dose) among children aged 9–59 months was 76.0% (CI 95%: 70.6–81.3; Table 2).

**Table 2:** Measles vaccination coverage, children 9 to 59 months old, Aketi Health Zone, Province Orientale, Democratic Republic of Congo, October 2013.

	Number vaccinated	Proportion	95% CI	Design Effect	ICC*
***According to vaccination card ***					
At least one dose	121	11.3%	6.1–16.4	7.3	0.244
Two doses	57	5.3%	1.8–8.8	6.7	0.221
***According to parental recall***					
At least one dose	693	64.7%	57.6–71.8	6.2	0.202
Two doses	561	52.4%	43.4–61.4	9.0	0.310
***According to vaccination card or by parental recall***					
At least one dose	814	76.0%	70.6–81.3	4.4	0.132
Two doses	618	57.7%	49.2–66.1	8.2	0.279
***Missing data***						131				
***Total***						1202				

* ICC is calculated as follows: (design effect– 1)/(average cluster size– 1)

### Risk factors for measles mortality

**Fig 2:**
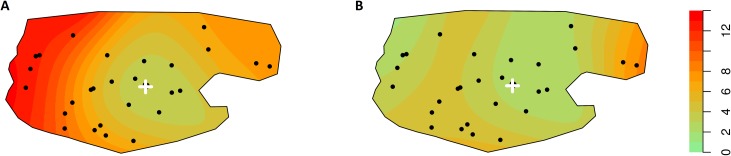
**Geospatial representation of CFR from December 25th, 2012 to February 28th 2013 (A), and from March 1st, 2013 to October 9th, 2013 (B) , Aketi Health Zone, Province Orientale, Democratic Republic of Congo.** The white cross is at the location of the central hospital of Aketi. The scale represent the CFR expressed in %. The 40 dots represent the cluster locations.

CFR was 6% during the period before 1 March and 3% after (Table 3). CFR was higher in the areas of the periphery farthest away from Aketi Hospital during the period before 1 March (Fig 2), after which time (when the intervention package became fully operational) CFR was lower and broadly heterogeneous throughout the HZ.

**Table 3:** Univariate analysis of factors associated with measles case fatality, Aketi Health Zone, Province Orientale, Democratic Republic of Congo, December 2012- October 2013.

Risk factor	Suspected cases	Deaths	CFR (%)†	Unadjusted RR	95% CI
Age at time of illness					
0–11 months	111 (10%)	9	8	7.1	1.5–33.8
12–23 months	153 (14%)	6	4	6.2	1.4–26.7
5–15 years	349 (31%)	9	3	2.4	0.5–11.4
>15 years	176 (16%)	2	1	Reference	
Unknown	4 (0%)	0	0		
Total	1131				
Received medical care					
No	123 (11%)	13	11	3.0	1.5–57
Yes	1008 (89%)	35	3	Reference	
Date of disease onset					
Before 1 March 2013	370 (33%)	24	6	2.1	1.2–3.8
After 1 March 2013	761 (46%)	24	3	Reference	
Sex					
Female	609 (54%)	28	5	Reference	
Male	521 (46%)	20	4	0.9	0.5–1.6
Unknown	1 (0%)	0	0		
At least one other case in household					
No	309 (27%)	20	6	1.9	1.0–34
Yes	822 (73%)	28	3	Reference	
Household size					
≤7 members	679 (60%)	25	4	Reference	
>7 members	452 (40%)	23	5	1.4	0.8–2.6
Distance to Aketi Hospital					
< = 30 km	438 (39%)	11	3	Reference	
>30 km	693 (61%)	37	5	2.2	1.0–4.7
Distance to nearest health‡ center (per 10 km)	1131			2.2	1.1–4.4
Measles vaccination history (children aged 9–59 months only), by vaccination card or parental recall					
No doses	85 (16%)	15	18	Reference	
One dose	106 (20%)	6	6	0.3	0.1–0.9
Two doses	307 (59%)	9	3	0.2	0.1–0.3
unknown	23 (4%)	2	9		
Total	521				

†CFR is calculated as follows: Deaths / Suspected cases

‡Distance by footpath treated as a continuous variable from the center of the villages where the cluster was located to the nearest health center or health post.

Among all suspected measles cases, younger age at time of illness, not receiving medical care in a health structure, being the only case in a household, disease onset before March 1, 2013 , and increased distance to the hospital and nearest health center were statistically significantly associated (at the 5% level) with a higher relative risk of dying (Table 3). For children aged 9 to 59 months, having received at least one dose and at least two doses of measles vaccine was protective against dying.

Clusters further away from the central hospital of the Health Zone were also more likely to be further away from a health center, resulting in a high correlation coefficient between these two variables (Pearson correlation coefficient = 0.4); therefore we included distance to the hospital in, and excluded distance to the nearest health center from, the multivariate model (the alternative option, where we included distance to the nearest healh centre is shown in the S1 File).

In the multivariate analysis, younger age was significantly associated with higher case fatality (with children under 1 year being at highest risk of dying). Not having received any form of health care was also strongly associated with a higher risk of death (Table 4). By introducing an interaction term between distance and disease onset ,we see a three folds higher risk for those living 30 km away from the hospital before March 1st compared to after March 1st (RR = 3.0, P = 0.001), but we do not see such evidence for people living within 30 km from the hospital (RR = 0.63 , P = 0.473). After march 1st , the risk is similar whatever the distance to the hospital is (RR = 1.2, P = 0.635) (details on the CFR before and after March 1st can be found in S1 File). Among children aged 9–59 months reported to have had measles during the recall period, having received two doses of measles vaccine was protective against dying (RR = 0.2, p = <0.001); a single dose was also protective but did not reach statistical significance (RR = 0.3, p = 0.054).

**Table 4:** Multivariate regression analysis of factors associated to measles fatality, Aketi Health Zone, Province Orientale, Democratic republic of Congo, December2012- October 2013.

Risk factor	Suspected Cases	Deaths	Adjusted RR	95% CI
Age at time of illness				
0–11 months	111	9	8.6	1.8–39.9
12–23 months	153	6	4.0	0.8–19.8
24–59 months	338	22	5.5	1.3–23.5
5–15 years	349	9	2.3	0.5–10.5
>15 years	176	2	Reference	
Received medical care				
No	123	13	3.2	1.6–6.2
Yes	1004	35	Reference	
Date of disease onset for cases living at a distance to Aketi hospital < = 30km				
Before 1 March 2013	177	4	0.63	0.2–2.2
After 1 March 2013	261	7	Reference	
Date of disease onset for cases living at a distance to Aketi hospital > 30km				
Before 1 March 2013	193	17	3.0	1.5–5.9
After 1 March 2013	500	20	Reference	
Distance to Aketi Hospital After 1 March 2013				
< = 30 km	261	7	Reference	
>30 km	500	17	1.2	0.5–3.1
Measles vaccination history (children aged 9–59 months only), by vaccination card or parental recall				
No doses	85	15	Reference	
One dose	106	6	0.3	0.1–0.9
Two doses	307	9	0.2	0.1–0.4

## Discussion

### Limitations

There are potential limitations to these results. We assumed that the improvement of access to quality health care led to the overall improvement in, and reduction of spatial associations with, CFR. However, other unmeasured factors, such as the change in road conditions, an increase awareness of the measles epidemic in remote area may have played a role. As with any retrospective survey, the results are subject to misclassification. Death is not easily forgotten, but individual symptoms of a disease are more difficult to remember. Dates are difficult to remember, for cases that occurred end of February beginning of March, it might have been difficult to accurately classify the measles case and measles death before or after 1^st^ of March. However, a calendar of events was used by the surveyors to minimize this risk. Numerous studies have shown the lack of reliability of vaccination cards and parental recall to estimate vaccination coverage [15][16].

The EPI method was used for secondary sampling, this method use a random approach to select the first household, the next households are chosen by proximity. This might have potentially leaded to a bias in case identification and possibly CFR determination. Misclassification bias could have influenced our results, as other diseases such as rubella can have similar symptoms to measles; however, laboratory confirmation of measles was done before the epidemic was declared, and the high CFR excludes rubella as a cause of the epidemic.

### Measles epidemic can still have a high burden

Our survey describes a large measles epidemic, in which the overall and under 5 AR was very high. The AR estimated in the survey was larger than the AR estimated using only cases notified in the surveillance system (10.4%), which is unsurprising given that under-reporting of infectious diseases is common when access to health care is limited[17][18][19][20]. Few epidemics reported in the literature have had such high ARs, and those that have been reported were generally in smaller populations than Aketi [18] [21][22].

The estimated measles CFR is comparable to that reported in similar community-based surveys [23], and is much higher than in industrialized countries [24]. It is also much larger than reported by the surveillance system, most likely due to an under-reporting of those measles deaths occurring in the community [25][26]. In the entire HZ, we estimated 798 deaths attributable to measles (95%CI: 520–1080) during the epidemic.

### Measles fatality in children reduced while access to quality health care, decentralization and effective referral increased

As reported in other surveys [23][26][27][28], younger age was associated with higher relative risk of mortality (though, the higher relative risk of mortality was not statistically significant in children aged 12–23 months), and not receiving treatment in a health structure was associated with almost a threefold higher relative risk of mortality. We found that prior vaccination was significantly associated with a lower risk of measles mortality, as reported elsewhere [23]. Even when other factors were taken into account (treatment received and age), the risk of dying was three fold higher for the people living 30 km away from the hospital before March 2013, in contrast this risk did not change significantly for people living close to the hospital. This occurred concurrently to the reinforcement of access to quality health care all over the health zone (see utilization of health care in S1 File) which assured free care for all suspected measles cases, conducted active case finding, and provided free referral for identified cases anywhere in the health zone to the hospital. Among other activities, the team set up an innovative referral system (community health workers referred cases using any motorbike available in the area; the motorbike drivers would then be reimbursed after arrival to the hospital) assured equity in access to health care for severe case. The study was not designed to estimates the impact of the different component of this comprehensive case management, further research could help to estimates the attributable impact of the different intervention.

### Prevention and control of the epidemic was not effectively implemented

Our estimate of one-dose measles-containing vaccine (MCV) coverage (either in the routine immunization or during SIA) among children aged 9–59 months was considerably below the threshold necessary to provide herd immunity [29]. As shown in other studies in DRC, the resurgence was likely caused by an accumulation of unvaccinated, measles-susceptible children due to low MCV coverage[30], this is likely the main cause of the epidemic.

The ORI started in May three weeks after the declaration of the epidemics. The number of cases treated for measles in health facilities started to increase in January, while the survey suggest that the number of cases in January in the community was already high. The epidemic curve both from the surveillance system and from the survey (S1 File) suggest that the ORI was implemented at the tail end of the epidemic, long after the epidemic was declared. ORI implemented earlier in the outbreak (January or February) , would have likely averted many of the 798 death that are attributable to this measles epidemic. It is unclear to the investigators if problems in the detection, notification or confirmation of the measles cases as well as other factor could have been the causes of the delayed epidemic declaration. The delayed ORI almost certainly contributed to the high AR—as has been shown in various other settings, epidemics often end soon after the introduction of ORI [31][32][33].

Lower than expected vaccine effectiveness has recently been found elsewhere in DRC [34]. This may have played a role in the size of this outbreak. This should be investigated further to understand what role, if any, it played.

## Conclusion

A third of the children under 5 years old were affected by measles during the large 2013 epidemic in Aketi HZ. The epidemic response was delayed, and ORI only took place after the epidemic peak. But we provide evidence suggesting that a comprehensive case management approach, including access to decentralized free health care and readily available referrals, reduced fatality during this epidemic in rural, inaccessible resource-poor setting.

## Supporting information

S1 File Supporting information. (DOCX)
