# Supplementary material for: Risk factors for measles mortality and the importance of decentralized case management during an unusually large measles epidemic in eastern Democratic Republic of Congo in 2013
Source: PLoS One. 2018 Mar 14;13(3):e0194276. doi: 10.1371/journal.pone.0194276 (PMC5851624; doi:10.1371/journal.pone.0194276)
Supplement: S1 File — (DOCX) [file pone.0194276.s001.docx]

Risk factors for measles mortality and the importance of decentralized case management during an unusually large measles epidemic in eastern Democratic Republic of Congo in 2013

*Technical Appendix*

Contents

[1 Additional key features of the study population 2](#_Toc503879701)

[2 Attack rates 5](#_Toc503879702)

[3 Measles vaccination coverage 6](#_Toc503879703)

[4 Utilization of health care services 7](#_Toc503879704)

[5 Stata syntax used for the multilevel model 8](#_Toc503879705)

List of Figure

[Figure 1. Measles Cases Fatality Ratio by Distance to the Aketi HZ central hospital , before and after March 1^st^ 2013 for every clusters of the sample, Aketi Health Zone, Province Orientale, Democratic Republic of Congo, January to September 2013 4](#_Toc503879650)

[Figure 2. Number of suspected measles cases by age at the time of the survey in the sample population, Aketi Health Zone, Province Orientale, Democratic Republic of Congo, October 2013 5](#_Toc503879651)

[Figure 3. Number of suspected measles cases by Month of disease in the sample population, Aketi Health Zone, Province Orientale, Democratic Republic of Congo, October 2013 6](#_Toc503879652)

List of Tables

[Table 1. Number of individuals by age group in the sample, Aketi Health Zone, Province Orientale, Democratic Republic of Congo, October 2013 2](#_Toc503879116)

[Table 2. Demographic description of the sample population, Aketi Health Zone, Province Orientale, Democratic Republic of Congo, October 2013 2](#_Toc503879117)

[Table 3. Description of the evolution of the sample population size during the recall period, Aketi Health Zone, Province Orientale, Democratic Republic of Congo, October 2013 2](#_Toc503879118)

[Table 4. Number of individuals, measles cases, measles deaths, and distance to the hospital by cluster, Aketi Health Zone, Province Orientale, Democratic Republic of Congo, October 2013 2](#_Toc503879119)

[Table 5. Measles Cases Fatality Ratio before and after March 1^st^, 2013 categorized by distance to the Aketi HZ central hospital below or over 30 km, Aketi Health Zone, Province Orientale, Democratic Republic of Congo, January to September 2013 4](#_Toc503879120)

[Table 6. Measles attack rate by age, 25 December 2012 to 9 October 2013, Aketi Health Zone, Province Orientale, Democratic Republic of Congo 4](#_Toc503879121)

[Table 7. Measles vaccination coverage by routine Immunization and supplementary immunization activities (SIA), children 9 to 59 months old, Aketi Health Zone, Province Orientale, Democratic Republic of Congo, October 2013 6](#_Toc503879122)

[Table 8. Utilization of health Care Services among suspected measles cases of the sample population, Aketi Health Zone, Province Orientale, Democratic Republic of Congo, October 2013 7](#_Toc503879123)

[Table 9. Multivariate regression analysis of factors associated to measles fatality, Aketi Health Zone, Province Orientale, Democratic republic of Congo, December2012- October 2013 7](#_Toc503879124)

# Additional key features of the study population

Table 1. Number of individuals by age group in the sample, Aketi Health Zone, Province Orientale, Democratic Republic of Congo, October 2013

| **Age group** | **Female** | **Male** | **Total** | **%** |
| --- | --- | --- | --- | --- |
| <5 years | 766 | 716 | 1 482 | 18.4 |
| 5–9 years | 660 | 671 | 1 331 | 16.5 |
| 10–14 years | 440 | 517 | 957 | 11.9 |
| >14 years | 2,250 | 2,034 | 4,284 | 53.2 |
| **Total** | 4,116 | 3,938 | 8,054 |  |

Table 2. Demographic description of the sample population, Aketi Health Zone, Province Orientale, Democratic Republic of Congo, October 2013

| **Item** | **Value** |
| --- | --- |
| Number of households included | 1,424 |
| Number of individuals included | 8,054 |
| Proportion of children <5 years | 18.40% |
| Proportion of children <15 years | 46.50% |
| Male:female ratio | 0.95 |
| Average household size | 5.7 |

Table 3. Description of the evolution of the sample population size during the recall period, Aketi Health Zone, Province Orientale, Democratic Republic of Congo, October 2013

| **Item** | **Number** |
| --- | --- |
| Individual in the household at the start of the recall period | 7,661 |
| Departure during the recall period | 175 |
| Death during the recall period | 122 |
| Individual in the household during the whole recall period | 7,364 |
| Individuals added to the household during the recall period | 436 |
| Newborns | 244 |
| Newcomers | 192 |
| Individual in the household at the day of the survey | 7,800 |
|  |  |
| Estimated mid-term population* | 7730 |
| *Individuals in the household on the day of the survey plus half of the departures and the deaths minus half of the newcomers and newborns. | |

Table 4. Number of individuals, measles cases, measles deaths, and distance to the hospital by cluster, Aketi Health Zone, Province Orientale, Democratic Republic of Congo, October 2013

| **Cluster** | **Individuals** | **Measles cases** | **Measles deaths** | **Distance to hospital** |
| --- | --- | --- | --- | --- |
| ***1*** | 259 | 50 | 5 | 60 |
| ***2*** | 171 | 23 | 0 | 55 |
| ***3*** | 197 | 35 | 4 | 53 |
| ***4*** | 206 | 26 | 1 | 32 |
| ***5*** | 197 | 27 | 2 | 40 |
| ***6*** | 166 | 34 | 2 | 61 |
| ***7*** | 201 | 14 | 2 | 83 |
| ***8*** | 126 | 21 | 0 | 30 |
| ***9*** | 217 | 24 | 4 | 30 |
| ***10*** | 243 | 30 | 0 | 6 |
| ***11*** | 241 | 30 | 1 | 4 |
| ***12*** | 220 | 22 | 1 | 4 |
| ***13*** | 147 | 11 | 0 | 2 |
| ***14*** | 223 | 21 | 0 | 4 |
| ***15*** | 240 | 55 | 0 | 4 |
| ***16*** | 231 | 20 | 0 | 4 |
| ***17*** | 266 | 34 | 0 | 4 |
| ***18*** | 212 | 44 | 3 | 51 |
| ***19*** | 201 | 14 | 0 | 32 |
| ***20*** | 205 | 7 | 0 | 20 |
| ***21*** | 162 | 29 | 0 | 47 |
| ***22*** | 175 | 45 | 0 | 42 |
| ***23*** | 215 | 25 | 1 | 4 |
| ***24*** | 190 | 47 | 1 | 84 |
| ***25*** | 217 | 27 | 0 | 24 |
| ***26*** | 142 | 15 | 0 | 35 |
| ***27*** | 163 | 13 | 3 | 62 |
| ***28*** | 181 | 19 | 2 | 19 |
| ***29*** | 183 | 3 | 0 | 16 |
| ***30*** | 188 | 31 | 1 | 4 |
| ***31*** | 205 | 25 | 0 | 1 |
| ***32*** | 230 | 33 | 1 | 2 |
| ***33*** | 215 | 33 | 1 | 60 |
| ***34*** | 210 | 17 | 2 | 65 |
| ***35*** | 203 | 26 | 2 | 87 |
| ***36*** | 180 | 28 | 1 | 112 |
| ***37*** | 272 | 63 | 1 | 105 |
| ***38*** | 218 | 43 | 2 | 75 |
| ***39*** | 180 | 27 | 3 | 175 |
| ***40*** | 199 | 40 | 2 | 142 |

Figure 1. Measles Cases Fatality Ratio by Distance to the Aketi HZ central hospital , before and after March 1^st^ 2013 for every clusters of the sample, Aketi Health Zone, Province Orientale, Democratic Republic of Congo, January to September 2013

Table 5. Measles Cases Fatality Ratio before and after March 1^st^, 2013 categorized by distance to the Aketi HZ central hospital below or over 30 km, Aketi Health Zone, Province Orientale, Democratic Republic of Congo, January to September 2013

|  |  | **Suspected Cases** | **Death** | **CFR** | **CI 95 %** |
| --- | --- | --- | --- | --- | --- |
| ***Before March 1st*** | |  |  |  |  |
|  | Distance to Aketi Hospital <= 30 km | 177 | 4 | 2.2% | [-2.2 - 6.8] |
|  | Distance to Aketi Hospital > 30 km | 193 | 20 | 10.4% | [6.0- 14.6] |
| ***After March 1st*** | |  |  |  |  |
|  | Distance to Aketi Hospital <= 30 km | 261 | 7 | 2.7% | [0.7- 4.6] |
|  | Distance to Aketi Hospital > 30 km | 500 | 17 | 3.4% | [1.8- 4.9] |

# Attack rates

Table 6. Measles attack rate by age, 25 December 2012 to 9 October 2013, Aketi Health Zone, Province Orientale, Democratic Republic of Congo

| **Age** | **Measles cases** | **Attack rate (%)** | **95% CI** | **Design effect** |
| --- | --- | --- | --- | --- |
| 0–11 months | 53 | 14.9 | 8.2–21.6 | 3.3 |
| 12–23 months | 104 | 45.6 | 36.1–55.0 | 2.1 |
| 24–59 months | 362 | 40.2 | 35.6–44.9 | 2.1 |
| 5–15 years | 419 | 18.3 | 14.9–21.7 | 4.7 |
| Unknown | 4 | - | - | - |
| >15 years | 189 | 4.4 | 3.1–5.6 | 4 |
| **Total** | **1,131** | **14.0** | **12.2–15.8** | **5.8** |

0

50

100

150

0

5

10

15

20

25

30

35

40

45

50

55

60

65

70

75

80

Age


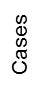


Figure 2. Number of suspected measles cases by age at the time of the survey in the sample population, Aketi Health Zone, Province Orientale, Democratic Republic of Congo, October 2013

0

50

100

150

200

250

Feb.

Jan.

Mar.

Ap.

May

Jun.

Jul.

Aug.

Sept.r

Month in 2013


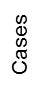


Figure 3. Number of suspected measles cases by Month of disease in the sample population, Aketi Health Zone, Province Orientale, Democratic Republic of Congo, October 2013

# Measles vaccination coverage

Table 7. Measles vaccination coverage by routine Immunization and supplementary immunization activities (SIA), children 9 to 59 months old, Aketi Health Zone, Province Orientale, Democratic Republic of Congo, October 2013

|  |  | **Number** | **Proportion** | **95% CI** | **Design effect** |
| --- | --- | --- | --- | --- | --- |
| ***Routine*** |  |  |  |  |  |
|  | With document | 89 | 11.20% | 5.6–16.8 | 6.3 |
|  | Parental recall | 522 | 65.70% | 56.1–75.3 | 6.5 |
|  | Not vaccinated | 183 | 23.00% | 15.7–30.4 | 8.4 |
|  | Missing data | 105 |  |  |  |
| ***SIA*** |  |  |  |  |  |
|  | With document | 65 | 8.10% | 3.9–12.3 | 4.9 |
|  | Parental recall | 551 | 68.90% | 60.1–77.6 | 7.3 |
|  | Not vaccinated | 184 | 23.00% | 14.5–31.4 | 8.4 |
|  | Missing data | 99 |  |  |  |
| ***Routine and/or SIA*** |  |  |  |  |  |
|  | At least one document | 109 | 13.30% | 7.7–18.8 | 6 |
|  | Parental recall | 601 | 73.40% | 65.3–81.4 | 7.1 |
|  | Not vaccinated | 109 | 13.30% | 7.6–19.0 | 5.7 |
|  | Missing data | 80 |  |  |  |

# Utilization of health care services

Table 8. Utilization of health Care Services among suspected measles cases of the sample population, Aketi Health Zone, Province Orientale, Democratic Republic of Congo, October 2013

|  |  |  | **Utilization of health faclities (%)** | | |
| --- | --- | --- | --- | --- | --- |
|  |  | N | Hospital | Health Centre only | all |
| **Before March 1st** | |  |  |  |  |
|  | Suspected measles Case living 30 km or less from Aketi | 177 | 35 (20%) | 101(57%) | 136 (77%) |
|  | Suspected measles Case living more than 30 km from Aketi | 193 | 43 (22%) | 121(62%) | 164(85%) |
|  | All suspected measles Case |  |  |  |  |
|  |  |  |  |  |  |
| **After March 1st** | |  |  |  |  |
|  | Suspected measles Case living 30 km or less from Aketi | 261 | 104(40%) | 143(55%) | 247(95%) |
|  | Suspected measles Case living more than 30 km from Aketi | 500 | 167(33%) | 294 (59%) | 461(92%) |
|  | All suspected measles Case |  |  |  |  |

Table 9. Multivariate regression analysis of factors associated to measles fatality, Aketi Health Zone, Province Orientale, Democratic republic of Congo, December2012- October 2013

| **Risk factor** | | | | **Cases** | **Deaths** |  | **Adjusted RR** | | | **95% CI** | | ***p*** |  |  |
| --- | --- | --- | --- | --- | --- | --- | --- | --- | --- | --- | --- | --- | --- | --- |
| Age at time of illness | | | |  |  |  |  | | |  | |  |  |  |
|  | 0-11 months | | | 111 | 9 |  | 8.6 | | | 1.8-39.9 | | 0.003 |  |  |
|  | 12-23 months | | | 153 | 6 |  | 4.0 | | | 0.8-19.8 | | 0.094 |  |  |
|  | 2-4 years | | | 338 | 22 |  | 5.5 | | | 1.3-23.5 | | 0.022 |  |  |
|  | 5-15 years | | | 349 | 9 |  | 2.3 | | | 0.5-10.5 | | 0.300 |  |  |
|  | >15 years | | | 176 | 2 |  | Reference | | |  | |  |  |  |
| Received medical care | | | |  |  |  |  | | |  | |  |  |  |
|  | No | | | 123 | 13 |  | 2.8 | | | 1.4-5.6 | | 0.003 |  |  |
|  | Yes | | | 1004 | 35 |  | Reference | | |  | |  |  |  |
| Date of disease onset | | | |  |  |  |  | | |  | |  |  |  |
|  | Before 1 March 2013 | | | 370 | 24 |  | 1.02 | | | 0.5-2.26 | | 0.961 |  |  |
|  | After 1 March 2013 | | | 757 | 24 |  | Reference | | |  | |  |  |  |
| Distance to the nearest health Centre* (per 10 km) | | | | 1127 |  |  | 1.1 | | | 0.5 -2.8 | | 0.768 |  |  |
|  |  | |  |  |  |  |  |  |  |  |  |  |  |  |
| Distance to the nearest health Centre (per 10 km) and Disease onset before March 1, 2013 | | | | 1127 |  |  | 3.2 | | | 1.0-10.6 | | 0.050 |  |  |
| Measles vaccination history (children aged 9-59 months only), by vaccination card or parental recall | | | | | | | |  |  | |  | | |  |
|  | No doses | | | 85 | 15 |  | Reference | | |  | |  |  |  |
|  | One dose | | | 106 | 6 |  | 0.4 | | | 0.1-1.1 | | 0.089 |  |  |
|  | Two doses | | | 307 | 9 |  | 0.2 | | | 0.1-0.4 | | <0.001 |  |  |
|  |  |  | |  |  |  | |  |  | |  | | |  |

*Distance by footpath treated as a continuous variable from the center of the villages where the cluster was located to the nearest health center or health post.

# Stata syntax used for the multilevel model

**Example for univariate analysis of age**

xi: xtmepoisson Death_Measles_Case i.Age_Group_measles if Measles==1, || Cluster:, covariance(independent) || Household:, covariance(independent) irr

**Example for multivariate analysis**

xtmepoisson Death_Measles_Case Received_Health_Care CaseBeforeMarch i i.Age_Group_measles HouseHold_size Distance_to Hospit_Sup30 Distance_to Health_center_sup10 if Measles==1, || Cluster:, covariance(independent) || Household:, covariance(independent) irr
